# Modified cardiopulmonary bypass with low priming volume for blood conservation in cardiac valve replacement surgery

**DOI:** 10.1186/s13019-023-02175-8

**Published:** 2023-02-02

**Authors:** Ke Yang, Honghao Huang, Ruiwu Dai, Jinbao Zhang, Xiaohong Wei, Feng Gao, Xiaochen Wu, Fan Wu, Siyi He, Mei Xin

**Affiliations:** 1 Department of Cardiovascular Surgery, General Hospital of Western Theater Command (Chengdu Military General Hospital), No. 270, Rongdu Rd, Jinniu District, Chengdu, 610036 China; 2 Present Address: College of Medicine, Southwest Jiaotong University, Chengdu, 610036 China; 3 General Surgery Center, General Hospital of Western Theater Command (Chengdu Military General Hospital), Chengdu, 610036 China

**Keywords:** Cardiopulmonary bypass, Low priming volume, Blood conservation, Cardiac surgery

## Abstract

**Background:**

The adverse effects of cardiopulmonary bypass during open cardiac surgery, including hemodilution, seem to be inevitable, especially for patients who generally have a relatively lower BMI with relatively small blood volumes. This study reports the modification and use of a cardiopulmonary bypass (CPB) system to reduce priming volume and hemodilution.

**Methods:**

This is a retrospective study of 462 adult patients who underwent cardiac valve replacement surgery from January 2019 to September 2021 at the General Hospital of Western Theater Command. The modified group consisted of 212 patients undergoing modified CPB. The control group included 250 patients receiving conventional CPB. Evaluated indices included fluid intake and output volumes during CPB, intraoperative indices related to CPB operation, usage of blood products during the peri-CPB period, and postoperative outcomes.

**Results:**

The modified group displayed a significant reduction in the crystalloid (200 mL vs. 600 mL, *P* < 0.05) and colloid priming volumes (450 mL vs. 1100 mL, *P* < 0.05), and ultrafiltration solution volume (750 mL vs. 1200 mL, *P* < 0.05). Furthermore, the modified group had a significantly lower rate of defibrillation (30.2% vs. 41.2%, *P* < 0.05). The intraoperative urine volume (650 mL vs. 500 mL, *P* < 0.05) and intraoperative hematocrit (Hct) (26% vs. 24%, *P* < 0.05) of the modified CPB group were also higher than in the control group. The modified group required a lower infusion volume of packed red blood cells (250 mL vs. 400 mL, *P* < 0.05) and lower infusion rates of packed red blood cells (17.9% vs. 25.2%, *P* < 0.05) and fresh frozen plasma (1.41% vs. 5.2%, *P* < 0.05). In addition, the modified group showed significantly improved indices related to postoperative recovery.

**Conclusions:**

The modified CPB system effectively conserves blood and shows noteworthy potential for application in cardiac valve replacement surgery.

**Supplementary Information:**

The online version contains supplementary material available at 10.1186/s13019-023-02175-8.

## Background

Cardiopulmonary bypass (CPB) remains an important tool for circulatory support in cardiac valve replacement surgery, but its adverse effects on blood seem to be inevitable, especially for patients with a lower BMI and relatively small blood volumes. Extreme hemodilution caused by large priming volumes decrease colloid osmotic pressure, resulting in the transfer of intravascular fluid to the tissue gap. The accumulation of extravascular fluid induces interstitial edema and corresponding organ dysfunction [1, 2]. In addition, due to damage caused by contact between blood and the artificial material in CPB circuitry and substantial bleeding during open chest procedures, patients usually develop severe intraoperative and postoperative anemia, which is associated with increased morbidity and mortality [3]. Although allogeneic red cell transfusions are performed on approximately 40–60% of patients undergoing cardiac valve replacement during hospitalization, growing evidence suggests an association between blood transfusion and unfavorable morbidity, mortality, and long-term outcomes after cardiac surgery [4–6]. This has led to the development of various blood conservation strategies during CPB in an attempt to reduce the demand for blood transfusion.

Traditional blood conservation efforts include a variety of techniques, such as preoperative anemia management [7], antifibrinolytic use [8], lowering of transfusion triggers [8], and topical hemostatic agents [9]. However, the problem of hemodilution caused by the priming solution in CPB remains. Recently, minimally invasive extracorporeal circulation (MiECC) has emerged as an attractive new CPB system for blood conservation. The features of MiECC include the absence of a venous reservoir, minimization of CPB circuits, optimization of the surface coating of components, usage of a centrifugal pump, and strategy for shed blood management [10]. These properties contribute to a low priming volume, an efficient blood-air interface, and a biocompatible coating system, thus attenuating hemodilution. This system reduces priming volume by approximately 30–50% and the infusion rate of different blood products by approximately 3–15%, as observed in a variety of randomized control trials [11, 12], meta-analyses [1, 13], and observational studies [14]. The blood conservation efficacy of MiECC was also supported by class IIa B evidence in the 2019 EACTS/EACTA/EBCP guidelines on CPB in adult cardiac surgery [10], indicating a promising platform for improving the safety of cardiac surgery.

Nevertheless, MiECC has not been extensively applied worldwide, owing to some unresolved issues, such as its relatively complex operation and high cost. Moreover, abandoning the venous reservoir is a controversial measure as while it decreases the priming volume, it further increases the difficulty of emboli management during CPB. For cardiac surgeries associated with higher bleeding rates, such as cardiac valve replacement surgery, the absence of a venous reservoir also leads to excessive intraoperative blood loss and increases the required volume of blood transfusion. More importantly, the above drawbacks may have greater impact on patients with a relatively lower BMI with relatively small blood volumes. Therefore, modifying conventional CPB (CCPB) may offer more practical value. Some pioneering studies have reported modifications on CCPB, such as the minimization of CPB circuits and the integration of arterial line filters, which have further improvements in performance specifications. As a result, priming volume was decreased by approximately 500 mL, which led to a red blood cell transfusion rate as low as 13.6% [15].

Inspired by these previous studies, we introduce a novel strategy for modifying the CCPB system to optimize blood conservation in adult cardiac valve replacement surgery through median sternotomy. We hypothesized that this modified CPB may reduce the priming volume of CPB devices, intraoperative hemodilution, and postoperative transfusions, all of which are favorable for intraoperative blood conservation and postoperative outcomes in patients undergoing cardiac valve replacement.

## Methods

### Study setting

Data were retrospectively collected from 462 adult patients who underwent cardiac valve replacement surgery from January 2019 to September 2021 at the General Hospital of Western Theater Command. The inclusion criteria were as follows: (1) age > 18 years; (2) patients with valvular heart disease diagnosed by cardiac color Doppler ultrasound; and (3) patients undergoing cardiac valve replacement surgery through median sternotomy in our department. The exclusion criteria were as follows: (1) patients who underwent emergency or redo cardiac surgery; (2) preoperative hematocrit (Hct) < 30; (3) patients requiring preoperative blood transfusion; and (4) patients requiring concurrent coronary artery bypass grafting or vascular surgery. The patients from January 2019 to June 2020 received conventional CPB during the surgery, while patients from July 2020 to September 2021 received modified CPB. After exclusion, 212 patients were included in the modified group. The control group consisted of 250 patients who met the same exclusion criteria. The patients in the control group were matched in terms of sex, age, weight, height, body mass index (BMI), preoperative Hct, diagnosis, surgical procedures, and concomitant surgeries distribution for comparisons with the modified group. This study was approved by the Institutional Ethical Review Board of the General Hospital of Western Theater Command (2020ky013).

### CPB system and procedures

Information regarding the reagents and materials used for the CPB system is provided in the Additional file 1.

#### The CCPB system

The CCPB system comprised roller pumps, an oxygenator, an arterial line filter, cardioplegia, and its perfusion circuit. The main pump tubing, a tubing connecting the venous reservoir with an oxygenator and passing through the main pump, was polyvinyl chloride (PVC)/silicone composite tubing with a total length of 210 cm, in which the diameter of the PVC and silicone sections were 10 mm and 12 mm, respectively. The arterial and venous ports of the oxygenator were connected with PVC tubing. The venous tubing was 12 mm in diameter and 135 cm in length, and the arterial tubing was 10 mm in diameter and 120 cm in length. The diameter of the tubing from the outlet end of the oxygenator artery to the arterial line filter was 10 mm and the length was 75 cm. Thus, the total length of the CCPB circuit was 540 cm. The CCPB system adopts gravity drainage mode, and the venous reservoir is 10 cm away from the ground. Histidine-tryptophan-ketoglutarate (HTK) solution was applied in the CCPB for cardioplegia. The single perfusion dose of cold HTK solution (4–8 °C) was 30 mL/kg. A suction tube was placed in the coronary sinus to remove the discharged fluid and prevent the HTK solution from entering the blood circulation. The perfusion pressure was maintained at 100 mmHg before cardiac arrest. After cardiac arrest, the perfusion pressure was reduced to 50 mmHg to ensure complete perfusion of the remaining solution. The total perfusion time was kept between 5 and 7 min. The perfusion circuit of cardioplegia included a cold filling device, a rolling pump, and a perfusion needle, which were connected by PVC/silicone tubing with a total length of 300 cm. In order to recover intraoperative bleeding as much as possible, the CCPB system was also equipped with left atrial suction and an intracardiac suction.

#### The modified CPB system

**Fig. 1:**
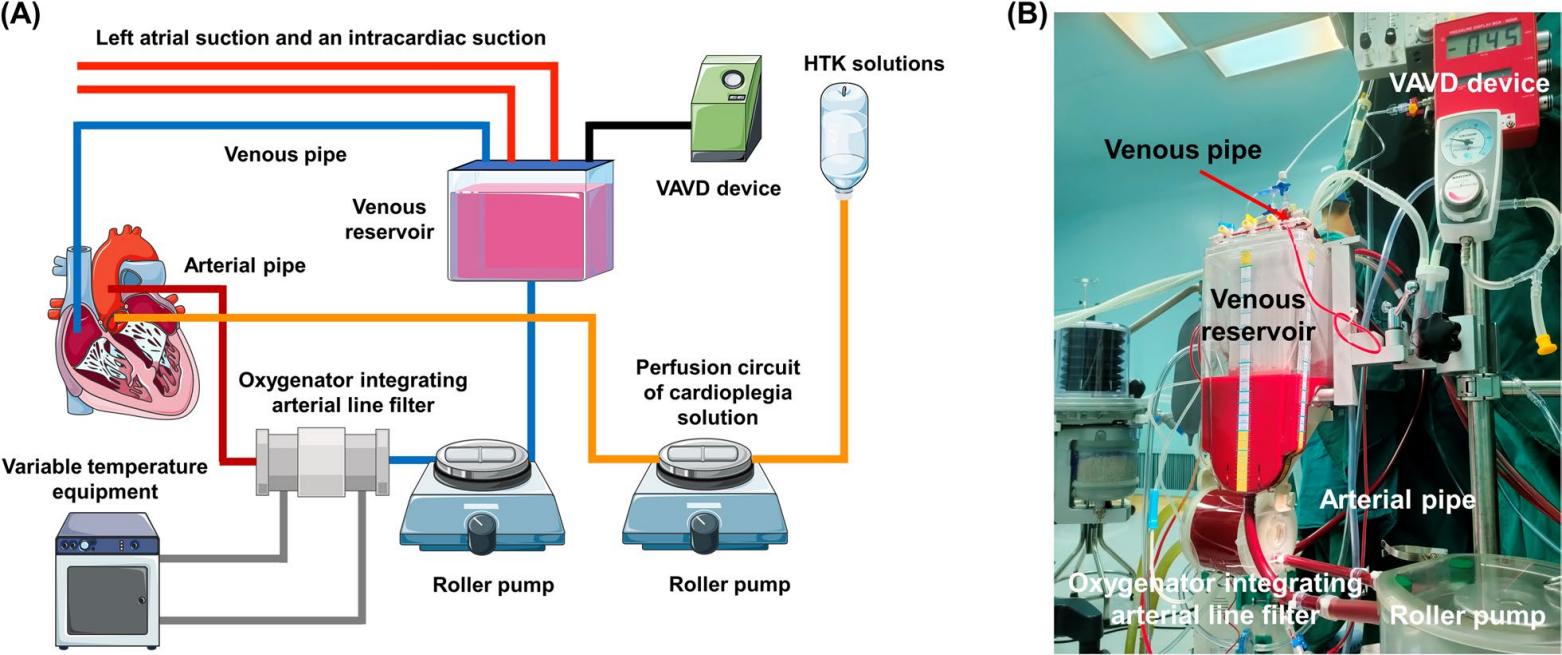
**A** Schematic description of the modified CPB system for cardiac valve replacement surgery through median sternotomy. **B** Images of the main components of the modified CPB system. CPB, VAVD and HTK refer to cardiopulmonary bypass, vacuum assist venous drainage and histidine-tryptophan-ketoglutarate, respectively

As shown in Fig. 1, the modified CPB system comprised roller pumps, an oxygenator integrating an arterial line filter, a vacuum-assisted venous drainage (VAVD) device, cardioplegia, and its perfusion circuit. The comparisons of detailed parameters between the modified CPB and CCPB system are listed in Table 1.

**Table 1:** Comparisons of the modified CPB system with the CCPB system

Parameters	CCPB system	Modified CPB system
CPB tubing	Main pump tubing: 210 cm	Main pump tubing: 80 cm
	Venous tubing: 135 cm	Venous tubing: 110 cm
	Arterial tubing: 120 cm	Arterial tubing: 100 cm
	Tubing from the outlet of the oxygenator artery to the arterial line filter: 75 cm	Total length: 290 cm
	Total length: 540 cm	Tubing diameter: approximately 10 mm (3/8 inch)
	Tubing diameter: approximately 10 mm (3/8 inch)	Priming volume: 228 mL
	Priming volume: 424 mL	
Oxygenator	Common oxygenator (RX25 type, Terumo)	Oxygenator integrating arterial line filter (FX25 type, Terumo)
	Position: 10 cm from the ground	Position: 80 cm from the ground
	Priming volume: 250 mL	Priming volume: 260 mL
Arterial line filter	Separate type	Integrated type
	Priming volume: 180 mL	
Ultrafiltration circuit	Priming volume: 70 mL	Priming volume: 70 mL
Venous reservoir	Priming volume: 576 mL	Priming volume: 192 mL
Assist device	–	VAVD device
*Assist operation*		
Retrograde autologous priming method	–	Yes (discharging 100 mL of priming solution)
CO_2_ flush procedure	–	Yes
Cardioplegia perfusion	Single HTK solutions perfusion	Single HTK solutions perfusion
Total priming volume	1500 mL	650 mL

CPB: Cardiopulmonary bypass; CCPB: conventional cardiopulmonary bypass; HTK: histidine-tryptophan-ketoglutarate; VAVD: vacuum assist venous drainage

Compared with the CCPB system, our modified CPB system featured the following improvements:

1. Shortening the CPB tubing lengths: To reduce priming volume, the modified CPB system featured a shortened main pump tubing of 80 cm, arterial tubing of 100 cm, and venous tubing of 110 cm. The total length of the system tubing was 290 cm, 250 cm shorter than that of the CCPB system.
2. Incorporating an arterial line filter into the oxygenator: The modified CPB system used an oxygenator that integrated an arterial line filter, and the position of the oxygenator was raised to 80 cm above the ground to minimize the length of the tubing between the oxygenator and the main pump (Fig. 1B).
3. Use of a VAVD device: To ensure adequate venous drainage, the modified CPB system featured a VAVD device added to the venous reservoir and continuously monitored venous end pressure.
4. Adopting the retrograde autologous priming method: To further reduce priming volume, a retrograde autologous priming step was added during CPB preparation. Deoxyepinephrine (5 µg) was first applied before the operation to maintain the patient's blood pressure above 100 mmHg. If systolic pressure fell below 50 mmHg, the retrograde autologous priming operation was aborted and the patients were excluded from the modified group. In this study, all 212 patients in modified group tolerated the retrograde autologous priming.
  1. the liquid storage bag was connected to the tee connection of the side branch circulation of the oxygenator. After this, the rolling pump was started to discharge the priming solutions in the venous reservoir into the liquid storage bag, and the pump was stopped when the liquid level of the venous reservoir was 100 mL.
  2. After successful aortic cannulation, the arterial clamp was opened, slowly making the patient's arterial blood retrograde, replacing the priming solutions in the arterial tube, and discharging the priming solutions in the arterial tube into the liquid storage bag from the tee connection of the collateral circulation of the oxygenator.
  3. After successful venous cannulation, the venous clamping forceps and VAVD device were opened, using negative pressure to discharge the priming solutions in the venous tubing into the venous reservoir, the venous tubing was clamped immediately after the blood filled the venous tubing, the VAVD device closed, and the rolling pump restarted to discharge the priming solutions in the venous reservoir into the liquid storage bag.
  4. Finally, the venous tubing and VAVD device were opened, a small portion of venous blood was drained into the venous reservoir, the rolling pump began to make the blood in the venous reservoir enter the main pump tubing, and the priming solutions discharged into the liquid storage bag to complete the entire operation.
5. Addition of the CO_2_ flush procedure to the CPB circuit before surgery: During the CPB system assembly, the tubing was flushed with CO_2_ for > 10 min to eliminate air in the device.

#### Anesthesia, surgical procedures, and CPB management strategies

All patients were treated with the same anesthetic induction and maintenance procedure, surgical approach, and CPB management strategy. The surgical details are described in the Additional file 1.

### Outcome measures

#### Fluid intake and output volumes during CPB

Four fluid intake and output volumes were measured during CPB: crystalloid priming volume (mL), colloid priming volume (mL), ultrafiltration solution volume (mL), and intraoperative urine volume (mL). The amount of fluid given prior to bypass was approximately 100 mL in both groups, the majority of which was the liquid volume of the narcotic drugs used.

#### Intraoperative indices related to CPB

The intraoperative indices related to CPB were measured, including cardiac surgery time (min), CPB operation time (min), cross-clamp time (min), net volume of infused cardioplegia (mL), rate of defibrillation (%), pre-CPB lactic acid (Lac) (mmol/L), 1-h intraoperative Lac (mmol/L), post-CPB Lac (mmol/L), pre-CPB Hct (%), 1-h intraoperative Hct (%), post-CPB Hct (%).

#### Usage of blood products during the peri-CPB period

Blood volume return from the CPB device (mL), the infusion volume of packed red blood cells (mL), and the infusion rate of different blood products (%) were measured. The peri-CPB period refers to the period from the beginning of CPB to hospital discharge, in which the amount of transfusion immediately after bypass or intraoperatively is also included. Blood volume return from the CPB device refers to the blood volume that remained in the extracorporeal circulation after bypass. The packed red blood cells were infused when the patient's Hct was less than 20% during surgery or less than 24% while in the intensive care unit (ICU). During the hemostasis process of surgery, Fresh frozen plasma (FFP) was infused when the anesthesiologist determined that the patient has hypotension caused by insufficient volume. In ICU, FFP was infused when the R and K values of thromboelastogram significantly increased and the MA and Mε values of thromboelastogram significantly decreased. In addition, the drainage volume of patients was considered to maintain effective circulation volume. Platelets were infused when the patient had obvious bleeding indication or the number of platelets was less than 30 × 10^9^/L.

#### Postoperative outcome indices

The 12-h postoperative Hct (%), 12-h postoperative Lac (mmol/L), incidence of complications, ICU results, and post-operative total length of stay were also collected.

### Statistical analysis

The Kolmogorov–Smirnov method was used to test the normality of the continuous variables. Normally distributed continuous variables were expressed as mean and standard deviation, and a *t*-test was used for the comparison between groups. Non-normally distributed continuous variables were expressed as median (interquartile range), and a nonparametric test was used for the comparison between groups. Categorical variables were expressed as frequencies and percentages. The ${\chi }^{2}$ test or Fisher’s exact probability method were used for intergroup comparison. IBM Statistical Package for the Social Sciences Statistics software (version 26.0) was used for the statistical analysis. All statistical tests were two-sided. A two-tailed *P* value below 0.05 was recognized as statistically significant for each test.

## Results

### Patient demographics and clinical characteristics

The modified group consisted of 212 patients undergoing modified CPB, while the control group included 250 patients receiving conventional CPB. No significant differences were observed between the two groups in terms of baseline demographic characteristics, including sex, age, weight, height, and BMI (Table 2). The median preoperative Hct levels were 41% and 42% in the modified and control group, respectively (*P* = 0.497). Most patients required valve replacement for rheumatic heart disease, but other indications included congenital valvular disease and degenerative valvular disease. All patients underwent aortic valve replacement, mitral valve replacement, or double valve replacement. Some patients underwent concomitant surgeries, such as concurrent tricuspid valve repair or left atrial plication. The two groups did not differ in their diagnoses, surgical procedures, and concomitant surgeries (Table 2).

**Table 2:** Patient demographics and clinical characteristics

Parameters	Control group (*n* = 250)	Modified group (*n* = 212)	*P* value
*Sex*, *n* (%)			
Male	161 (64.40)	141 (66.50)	0.076
Female	89 (35.60)	71 (33.50)	0.076
Age, years	49.39 ± 2.76	54.43 ± 2.46	0.126
Weight, kg	60 (53, 65)	60 (53, 65)	0.426
Height, cm	160 (155, 165)	160 (155, 165)	0.466
BMI, kg/m^2^	23.48 (20.96, 25.41)	22.82 (20.97, 25.08)	0.167
Preoperative Hct, %	42 (35, 49)	41 (36, 47)	0.497
EF, %	46 (40, 54)	44 (41, 56)	0.139
LVEDD, mm	41 (36, 45)	39 (37, 46)	0.245
*Diagnosis*, *n* (%)			
Rheumatic heart disease	159 (63.60)	140 (66.03)	0.438
Congenital valvular disease	12 (4.80)	13 (6.13)	0.345
Degenerative valvular disease	64 (25.60)	49 (23.11)	0.067
*Surgical procedures*, *n* (%)			
Double valve replacement	87 (34.80)	76 (35.85)	0.147
Aortic valve replacement	59 (23.60)	61 (28.77)	0.206
Mitral valve replacement	85 (34.00)	68 (32.08)	0.661
*Concomitant surgeries*, *n* (%)			
Tricuspid valve repair	187 (74.80)	163 (78.89)	0.602
Left atrial plication	19 (7.60)	21 (9.90)	0.380

BMI: Body mass index; Hct: hematocrit; EF: ejection fraction; LVEDD: left ventricular end-diastolic diameter. Non-normally distributed continuous variables are expressed as median (interquartile range)

### Fluid intake and output volumes during CPB

**Fig. 2:**
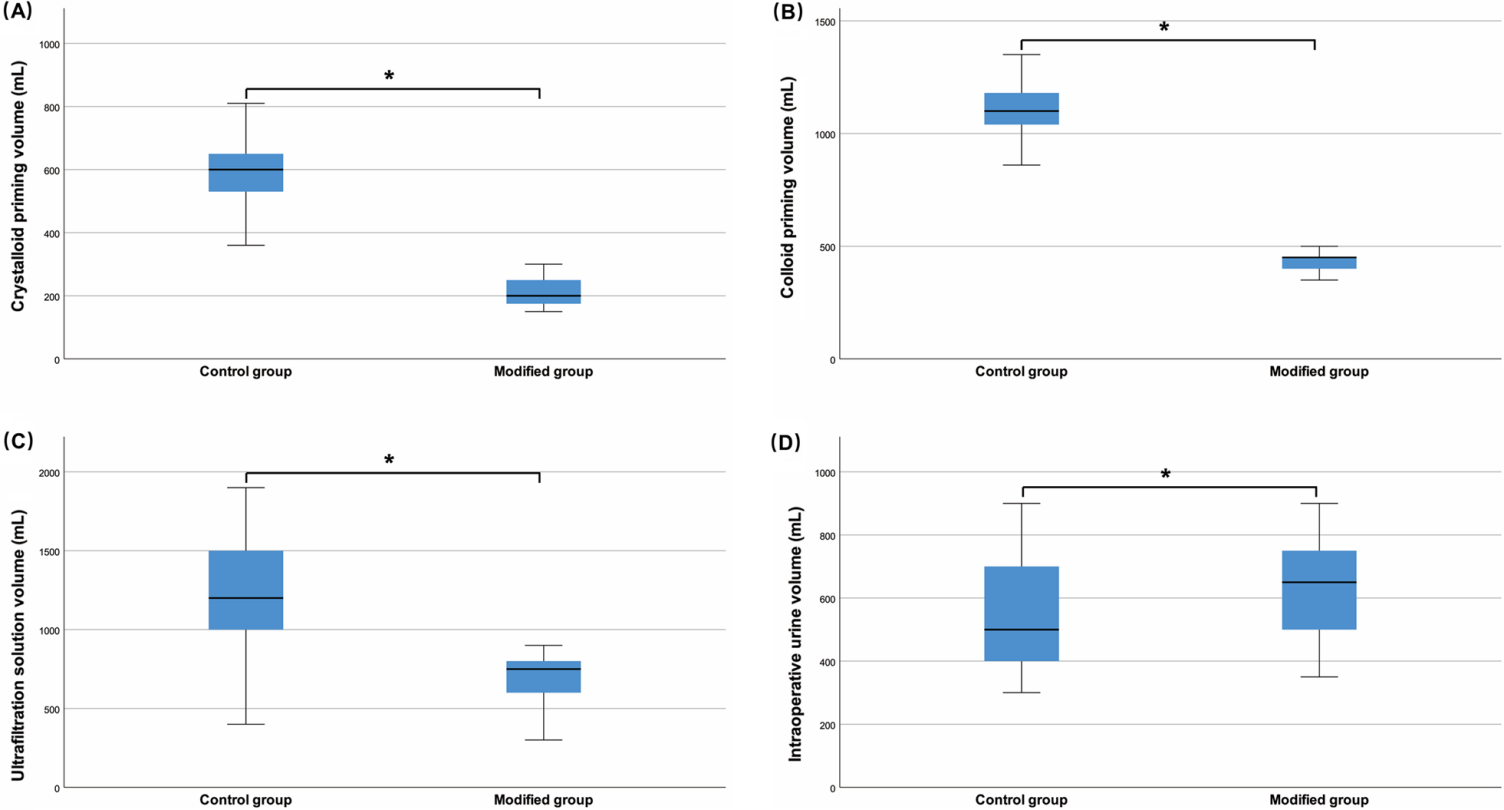
Fluid intake and output volumes, including crystalloid priming volume (**A**), colloid priming volume (**B**), ultrafiltration solution volume (**C**), and intraoperative urine volume (**D**) between control and modified groups. Error bars are standard error. * Refers to (*P* < 0.05). Non-normally distributed continuous variables are expressed as medians (interquartile range)

To investigate the influence of the modified CPB system on intraoperative blood conservation, fluid intake and output volumes were measured during CPB. Compared with the control group, the modified group had a significantly lower crystalloid priming volume (200 mL vs. 600 mL, *P* < 0.05) (Fig. 2A), colloid priming volume (450 mL vs. 1100 mL, *P* < 0.05) (Fig. 2B), and ultrafiltration solution volume (750 mL vs. 1200 mL, *P* < 0.05) (Fig. 2C). In addition, the intraoperative urine volume in the modified group was higher than that in the control group (650 mL vs. 500 mL, *P* < 0.05) (Fig. 2D).

### Intraoperative indices related to CPB

As shown in Table 3, the two groups did not differ in terms of cardiac surgery time, CPB operation time, or cross-clamp time (all *P* > 0.05). The modified group had a significantly higher net volume of infused cardioplegia (800 mL vs. 500 mL, *P* < 0.05) compared with the control group. The modified group had a significantly lower rate of defibrillation (30.2% vs. 41.2%, *P* < 0.05) compared with the control group, indicating better intraoperative myocardial protection. To evaluate whether intraoperative circulatory perfusion was appropriate, some key indices of arterial blood gas analysis were investigated. No significant differences in pre-CPB Lac, 1-h intraoperative Lac, post-CPB Lac, or pre-CPB Hct levels were observed between the two groups, whereas the modified group had significantly a higher 1-h intraoperative Hct (26% vs. 24%, *P* < 0.05) and post-CPB Hct (33% vs. 30%, *P* < 0.05).

**Table 3:** Intraoperative indices related to CPB between control and modified groups

Parameters	Control group (*n* = 250)	Modified group (*n* = 212)	*P* value
Cardiac surgery time (min)	218.45 ± 5.45	216.37 ± 5.98	0.145
CPB operation time (min)	112 (89, 135)	120 (95, 140)	0.052
Cross-clamp time (min)	76 (56, 102)	83 (63, 107)	0.114
Net volume of infused cardioplegia (mL)	500 (450, 650)	800 (500, 1100)	< 0.05
Rate of defibrillation, *n* (%)	103 (41.2)	64 (30.2)	< 0.05
Pre-CPB Lac (mmol/L)	0.7 (0.5, 1.3)	0.8 (0.5, 1.4)	0.361
Intraoperative 1-h Lac (mmol/L)	1.7 (1.4, 2.1)	1.6 (1.2, 2.1)	0.257
Post-CPB Lac (mmol/L)	2.4 (1.5, 2.8)	2.2 (1.4, 2.6)	0.157
Pre-CPB Hct (%)	41 (36, 52)	40 (35, 50)	0.073
Intraoperative 1-h Hct (%)	24 (21, 27)	26 (24, 30)	< 0.05
Post-CPB Hct (%)	30 (26, 35)	33 (29, 35)	< 0.05

CPB: Cardiopulmonary bypass; Hct: hematocrit. Non-normally distributed continuous variables are expressed as medians (interquartile range)

### Usage of blood products during the peri-CPB period

As shown in Table 4, there were no significant differences in blood volume return from the CPB device, indicating that the two systems may have a similar blood loss volume. However, the infusion volume of packed red blood cells in the control group outweighed the requirements of the modified group (400 mL vs. 250 mL, *P* < 0.05). With respect to the use of specific blood products, packed red blood cells were administered to 38 patients (17.9%) in the modified group versus 63 patients (25.2%) in the control group (*P* < 0.05). Only three patients (1.41%) received plasma in the modified group, compared with 13 control group patients (5.2%) (*P* < 0.05). There were no significant differences in platelet infusion rates between the two groups (*P* = 0.835).

**Table 4:** Usage of blood products during the peri-CPB period between control and modified groups

Parameters	Control group (*n* = 250)	Modified group (*n* = 212)	*P* value
Blood volume return from CPB device (mL)	600 (500, 700)	600 (500, 700)	0.218
Infusion volume of packed red blood cells (mL)	400 (300, 500)	250 (250, 375)	< 0.05
Infusion rate of packed red blood cells, *n* (%)	63 (25.2)	38 (17.9)	< 0.05
Infusion rate of fresh frozen plasma, *n* (%)	13 (5.2)	3 (1.41)	< 0.05
Infusion rate of platelets, *n* (%)	4 (1.6)	2 (0.94)	0.835

CPB: Cardiopulmonary bypass. Continuous variables not conforming to the normal distribution are expressed as median (interquartile spacing)

### Postoperative outcome indices

Table 5 displays the results of postoperative arterial blood gas analysis, the incidence of complications, and ICU outcomes. The modified group had a significantly higher 12-h postoperative Hct level than the control group (36% vs. 34%, *P* < 0.05). No significant differences in the 12-h postoperative Lac were observed between the two groups. Some patients in the two groups developed cerebrovascular events or reoperation for postoperative bleeding, but there were no significant differences in their incidence (0.94% vs. 1.20%, *P* = 1.000; 0.47% vs. 0.40%, *P* = 1.000, respectively). Additional procedures, such as continuous renal replacement therapy (CRRT), intra-aortic balloon pump (IABP) treatment, or extracorporeal membrane oxygenation (ECMO) treatment, were not required in the modified group. In the control group, two patients (0.80%) received IABP treatment, and one patient (0.40%) received ECMO treatment. There were no significant differences between the groups regarding CRRT (*P* = 1.000), IABP treatment (*P* = 1.000), and ECMO treatment (*P* = 1.000). With respect to ICU outcomes, the modified group required less ventilation time than the control group (18 h vs. 23 h, *P* < 0.05), but no significant differences in time spent in ICU were observed between the two groups (76 h vs. 79 h, *P* = 0.113). In addition, no significant differences in post-operative total length of stay were observed between the two groups (10 d vs. 11 d, *P* = 0.134).

**Table 5:** Postoperative outcome indices between control and modified groups

Parameters	Control group (*n* = 250)	Modified group (*n* = 212)	*P* value
Postoperative 12-h Hct (%)	34 (31, 37)	36 (32, 39)	< 0.05
Postoperative 12-h Lac (mmol/L)	1.7 (1.4, 2.1)	1.6 (1.2, 2.1)	0.051
Cerebrovascular events, *n* (%)	3 (1.20)	2 (0.94)	1.000
Reoperation for postoperative bleeding, *n* (%)	1 (0.40)	1 (0.47)	1.000
CRRT, *n* (%)	0 (0)	0 (0)	1.000
IABP, *n* (%)	2 (0.80)	0 (0)	1.000
ECMO, *n* (%)	1 (0.40)	0 (0)	1.000
Ventilation time (h)	23 (19, 35)	18 (14, 23)	< 0.05
ICU stay time (h)	79 (70, 89)	76 (69, 87)	0.113
Post-operative total length of stay, (d)	11 (9, 14)	10 (8, 14)	0.134

ICU: Intensive care unit; Hct: haematocrit; Lac: lactic acid; CRRT: continuous renal replacement therapy; IABP: intra-aortic balloon pump; ECMO: extracorporeal membrane oxygenation. Non-normally distributed continuous variables are expressed as median (interquartile range)

## Discussion

In this study, we reported the modification and use of a CPB system to reduce the priming volume and conserve blood in adult cardiac valve replacement surgeries. Briefly, the tubing length was shortened by 250 cm in our modified CPB, and approximately 850 mL less priming solution was required compared with the CCPB system. This decrease was more than the value reported in a similar modified-CPB study, which decreased the priming volume by approximately 500 mL [15, 16]. The lower priming volume observed in our study was attributed to the shortening of CPB tubing length, the integration of an arterial line filter into the oxygenator, the usage of a VAVD device, and the use of a retrograde autologous priming method. In particular, the priming volume of CPB tubing was calculated based on the tubing lengths and internal diameters. Thus, the reduction of 250 cm tubing length saved around 196 mL of priming volume. The integration of an arterial line filter into oxygenator saved 170 mL of priming volume. The usage of a VAVD device ensured that the venous blood could inflow into the venous reservoir, even when its priming liquid volume was lower than 200 mL, resulting in a reduction of 384 mL priming volume. Finally, the retrograde autologous priming method saved 100 mL in priming volume. The priming volume further decreased the fluid output volumes during CPB. Owing to the attenuated hemodilution from reduced priming solution, the removal volume of water-soluble components in ultrafiltration was reduced. Additionally, red blood cells and blood colloidal osmotic pressure remained at a relatively high level, the latter leading to a higher perfusion pressure in the kidney [17]. Therefore, patients in the modified group displayed a lower ultrafiltration solution volume and a higher intraoperative urine volume.

After decreasing the priming volume, the modified CPB system exhibited an attractive blood-conservation effect. Higher 1-h intraoperative Hct and 12-h postoperative Hct were observed in patients in the modified group. Additionally, the infusion rates of packed red blood cells and fresh frozen plasma decreased by 7.3% and 3.8%, respectively. These results indicate that the lower priming volume used in the modified CPB system reduced hemodilution. Successful attempts to minimize hemodilution and avoid blood transfusion by decreasing the priming volume have also been reported previously. A univariate analysis revealed that CPB circuit priming volume is a potentially modifiable risk factor for the need for blood transfusion in patients undergoing cardiac surgery with CPB [18]. A similar modified CPB investigation demonstrated that CPB priming volume reduction decreased the infusion rate of packed red blood cells and fresh frozen plasma in 1070 adult patients after cardiac surgeries by up to 13.6% and 6.5%, respectively [15]. Another retrospective study with 1724 adult patients receiving heart surgery also reported a reduction of approximately 5.3% in the transfusion of two or more units of packed red blood cells through decreasing CPB priming volume [19]. All these results were in line with that of our work. In addition to adult patients, neonates and children who underwent cardiac surgery with low CPB priming volumes displayed a substantial reduction in the requirements for blood transfusions [20–22], which may be attributed to the fact that the blood volume of low-weight patients is more sensitive to priming volume changes. Lower blood transfusion volumes could reduce the risk of transfusion-related complications and treatment costs. More importantly, compared with European and American individuals, Asian patients generally have a relatively lower BMI with relatively small blood volumes [23, 24], resulting in a higher probability of excessive hemodilution and blood transfusion during open-heart surgery. Therefore, the blood conservation of our modified CPB system renders it particularly advantageous in these settings.

Some intraoperative and postoperative outcome indices were also observed in this study. The differences between the groups were statistically non-significant regarding cerebrovascular events, treatment with IABP and ECMO, CRRT, and ICU stay time. A lower rate of defibrillation and shorter ventilation time were observed in patients in the modified group. Nevertheless, the shorter ventilation time seems to be due to the surgical management. Nowadays, patients with a problem-free course can be extubated on-table, using the fast-track concept. Similar ideas apply here for ICU stays.

Compared with previous work, the fabricated CPB system in this study included several improvements, but inevitably had limitations. Previous studies have found that adding the CO_2_ flush procedure contribute to a reduction of gaseous microemboli in CPB devices and corresponding improved neuropsychological outcomes [14, 25, 26]. Thus, we implemented the CO_2_ flush procedure before surgery in our modified CPB system. However, the differences between the two groups were not statistically significant regarding cerebrovascular events in our study. Potential biases of this result were the limited number of patients experiencing cerebrovascular events, the influence of CO_2_ flushing of the cardiac chambers during surgery, and the non-uniform administration technique for CO_2_ flushing. Therefore, the practical value of the CO_2_ flush is yet to be fully investigated. Our future work will aim to further explore its efficacy by expanding the number of patients and observation indicators regarding the remaining air in the CPB circuit, gaseous microemboli, and neuropsychological outcomes. Such studies may reveal other clinical improvements above blood conservation, such as cerebral protection. In addition, the lower incidence of defibrillations may suggest better myocardial protection, but the mechanism cannot be explained with a clamped aorta. We speculated that the lower incidence of defibrillations may be related to the improved blood conservation in the modified CPB group. Blood with a higher Hct may favor oxygen delivery to the myocardium in the early stage of CPB. However, we were unable to determine the detailed mechanisms in our study as there is no way to evaluate the impact of Hct on myocardial oxygen consumption during CPB. In addition, the lower incidence of defibrillations alone cannot conclude improved myocardial protection. Potential bias, such as the de-airing of the heart during surgery, should be excluded. More parameters representing myocardial protection should be observed in the future, including cardiac index or myocardial injury markers.

## Conclusions

In summary, a modified CPB system was fabricated and applied to adult cardiac valve replacement surgery. With the introduction of five optimization measures, the modified CPB system saved approximately 850 mL of priming solution and presented an attractive blood conservation effect compared with CCPB. Both the intraoperative and postoperative results revealed that the modified group exhibited a higher Hct, indicating less hemodilution. It also resulted in a reduction for the need of specific blood products, and infusion rates of packed red blood cells and fresh frozen plasma decreased by 7.3% and 3.8%, respectively. With the aid of the modified CPB system, a lower rate of defibrillation and shorter ventilation time were achieved. As a result, the clinical effectiveness of this CPB system, including myocardial protection, may be envisioned. These findings demonstrate that the modified CPB system described herein greatly conserves blood, with a noteworthy potential for application in cardiac surgery.

## Supplementary Information

**Additional file 1**: Supplementary material includes the reagents and materials used for the CPB system, the anesthetic procedure, surgical techniques, and CPB management strategies.

## Data Availability

The datasets generated during the current study are not publicly available but are available from the corresponding author on reasonable request.

## Abbreviations

CPB: Cardiopulmonary bypass
Hct: Hematocrit
MiECC: Minimally invasive extracorporeal circulation
CCPB: Conventional cardiopulmonary bypass
VAVD: Vacuum-assisted venous drainage
HTK: Tryptophan-ketoglutarate
CO_2_: Carbon dioxide
Lac: Lactic acid
ICU: Intensive care unit
BMI: Body mass index
EF: Ejection fraction
CRRT: Continuous renal replacement therapy
IABP: Intra-aortic balloon pump
ECMO: Extracorporeal membrane oxygenation
