# Supplementary material for: Modified cardiopulmonary bypass with low priming volume for blood conservation in cardiac valve replacement surgery
Source: J Cardiothorac Surg. 2023 Feb 2;18:56. doi: 10.1186/s13019-023-02175-8 (PMC9896670; doi:10.1186/s13019-023-02175-8)
Supplement: Supplementary file 1 — Additional file 1: Supplementary material includes the reagents and materials used for the CPB system, the anesthetic procedure, surgical techniques, and CPB management strategies. [file 13019_2023_2175_MOESM1_ESM.docx]

**Supplementary Material**

**Modified cardiopulmonary bypass with low priming volume for blood conservation in cardiac valve replacement surgery**

Ke Yang^1, #^, Honghao Huang^1,2 #^, Ruiwu Dai^2,3^, Jinbao Zhang^1^, Xiaohong Wei^1^, Feng Gao^1^, Xiaochen Wu^1^, Fan Wu^1^, Siyi He^1, *^, Mei Xin^1, *^

**Author affiliations:**

^1^ Department of Cardiovascular Surgery, General Hospital of Western Theater Command (Chengdu Military General Hospital), Chengdu, 610036, China.

^2^ College of Medicine, Southwest Jiaotong University, Chengdu, 610036, China.

^3^ General Surgery Center, General Hospital of Western Theater Command (Chengdu Military General Hospital), Chengdu, 610036, China.

**Correspondence to:**

*Mei Xin, Department of Cardiovascular Surgery, General Hospital of Western Theater Command (Chengdu Military General Hospital), No. 270, Rongdu Rd, Jinniu District, Chengdu, 610036, China. E-mail: [xinmei197212@163.com](mailto:xinmei197212@163.com). Tel: +8613981816035

*Siyi He, Department of Cardiovascular Surgery, General Hospital of Western Theater Command (Chengdu Military General Hospital), No. 270, Rongdu Rd, Jinniu District, Chengdu, 610036, China. E-mail: [hesiyi@vip.163.com](mailto:hesiyi@vip.163.com) Tel: +8613558837339

# Materials and methods

**Reagents and materials**

The roller pumps were from LivaNova Stockert SC heart-lung machine (Munich, Germany), while the oxygenator (RX25 type for CCPB system, FX25 type for modified CPB system) and ultrafiltration (HC11) were purchased from Terumo Cardiovascular Corporation (Tokyo, Japan). The arterial line filter (adult type), system tubing (adult type), arterial and venous cannulas (model dependent on the patient's weight), perfusion circuit (BII-1000), left atrial suction (adult type), and intracardiac suction (adult type) were obtained from Filar medical supplies Co., Ltd (Ningbo, China). The histidine-tryptophan-ketoglutarate (HTK) solutions (CUSTODIOL) were from DR. Franz Kohler Chemie Gmbh (Bensheim, Germany). The VAVD device (PM3500, VACUUM) and its pressure sensor (PDBR401791) were from Precision Medical Inc (Northampton, USA) and Medtronic (Minnesota, USA), respectively. The anesthetic drugs and priming solutions were from the General Hospital of Western Theater Command.

**Anesthesia induction, surgical procedures and CPB management strategies**

All patients fasted on water for 8 h before operation. After entering the operating room, the anesthesiologist monitored the patient's electrocardiogram and indwelling catheterization, opened the forearm venous access to monitor the central venous pressure, took mask oxygen inhalation, and monitored the arterial pressure by radial artery puncture under local anesthesia. Midazolam (0.2-0.3 mg/kg), propofol (1-2 mg/kg), etomidate (0.3 mg/kg), fentanyl (20-30 μg/kg), vecuronium (induction dose 0.1 mg/kg), and other drugs were used to induce and maintain the anesthesia.

Prior to CPB, crystal solution (lactate ringer solution), colloidal solution (hydroxyethyl starch), heparin, 5 % sodium bicarbonate, and 20 % mannitol were used as the priming solutions. All patients underwent a midline sternotomy and systemic heparinization performed by intravenous injection of 300 IU/kg heparin. The ascending aorta, as well as superior and inferior vena cava, were intubated to establish the CPB pathway. To begin CPB, the whole body was cooled. When the nasopharyngeal temperature reached 32 ℃, the ascending aorta was blocked, and cardiac arrest was induced by anterograde perfusion of the cardioplegic solution through the root of the aorta. The cardioplegic solution was aspirated via the coronary sinus during perfusion. During CPB, circulatory support was maintained by a 2.0-2.4 L/m^2^/min perfusion flow rate, 60-80 mmHg average arterial pressure, 75±10 % mixed venous oxygen saturation, 25-30 % target Hct, and 28-32 nasopharyngeal temperature. Ultrafiltration was used when the intraoperative Hct was lower than 25 %. Infusion of packed red blood cells was first considered when the intraoperative Hct was lower than 20 %. After intracardiac surgery, the left ventricular system gas was discharged, the ascending aorta was opened, and the coronary blood supply was restored. After heartbeat recovery, patients were gradually warmed up and taken off CPB. At the termination of CPB, the circuit volume was infused back into the patient.
